# Effects of unilateral/bilateral amputation of the ischiocavernosus muscle in male rats on erectile function and conception

**DOI:** 10.1186/s12610-021-00151-7

**Published:** 2022-01-05

**Authors:** Chengren Gou, Tong Liu, Zongping Chen, Zidong Zhou, Tao Song, Kaiyi Mao, Congcong Chen, Bo Chen

**Affiliations:** 1 Department of Urology, the Affiliated Hospital of Zunyi Medical University, Zunyi, 563000 China; 2 Department of Pediatric Surgery, the First Affiliated Hospital of Southwest Medical University, Luzhou, 646000 China; 3 Department of Urology, Eastern Hospital of Sichuan Provincial People’s Hospital, Chengdu, 610035 China

**Keywords:** Erectile dysfunction, Ischiocavernosus muscle amputation, Intracavernosal pressure, Male rats, Pregnancy rate, Dysfonction érectile, Amputation musculaire ischiocavernosus, Pression intracarvernosale, Rats mâles, Taux de grossesse

## Abstract

**Background:**

The ischiocavernosus muscle (ICM) encompasses a pair of short pinnate muscles attached to the pelvic ring. The ICM begins at the ischial tuberosity and ends at the crus of the penis while covering the surface of the crus. According to the traditional view, the contraction of the ICM plays an auxiliary role in penile erection. However, we have previously shown that the ICM plays an important role in penile erection through an indirect method of diagnosing erectile dysfunction (ED) caused by ICM injury by observing the infertility of paired female rats. Since intracavernosal pressure (ICP) is the current gold standard for diagnosing ED, this study aimed to amputate unilaterally/bilaterally the ICM to establish an ED model by detecting the ICP, recording the infertility of matching female rats, and comparing the two methods.

**Results:**

Forty sexually mature adult male rats were selected and randomly divided into the following groups: the control group (*n* = 10), sham operation group (*n* = 10), unilateral ischiocavernosus muscle (Uni-ICM) amputation group (*n* = 10), and bilateral ischiocavernosus muscle (Bi-ICM) amputation group (*n* = 10). Eighty female reproductive rats were randomly assigned to the above groups at a ratio of 2:1. We evaluated the time to conception for the paired female rats and the effects of unilateral/bilateral severing of the ICM on erectile function. The results showed that the baseline and maximum intracavernosal pressure (ICP) in the control group, sham operation group, Uni-ICM amputation group, and Bi-ICM amputation group were 17.44±2.50 mmHg and 93.51±10.78 mmHg, 17.81±2.81 mmHg and 95.07±10.40 mmHg, 16.73±2.11 mmHg and 83.49±12.38 mmHg, and 14.78±2.78 mmHg and 33.57±6.72 mmHg, respectively, immediately postsurgery. The max ICP in the Bi-ICM amputation group was lower than that in the remaining three groups (all *P*<0.05). The pregnancy rates were 100, 100, 90, and 0% in the control group, sham operation group, Uni-ICM amputation group, and the Bi-ICM amputation group, respectively. The pregnancy rate in the Bi-ICM amputation group was significantly lower than that in the remaining groups (all *P*<0.05). The time to conception was approximately 7–10 days later in the Uni-ICM amputation group than in the control and sham groups (all *P*<0.05).

**Conclusions:**

Male rats undergoing Bi-ICM amputation may develop permanent ED, which affects their fertility. In contrast, rats undergoing Uni-ICM amputation may experience transient ED.

## Résumé

**Introduction:**

Le muscle Ischiocavernosus (MIC) est une paire de muscles courts attachés à l’anneau pelvien. Elle commence à la tubérosité ischiale et se termine au crus du pénis tout en couvrant la surface de ce dernier. Selon la vision traditionnelle, la contraction du MIC joue un rôle auxiliaire dans l’érection pénienne. Cependant, nos travaux précédents ont montré qu’il joue un rôle important dans l’érection pénienne par une méthode indirecte de diagnostic de la dysfonction érectile (DE) induite par une blessure du MIC en observant l’infertilité des rats femelles appariés. Comme la pression intracaverneuse (PIC) est actuellement l’étalon de référence pour le diagnostic, cette étude visait à amputer unilatéralement/bilatéralement l’ICM pour établir un modèle de DE par détection de l’ICP, par détection de l’infertilité de rats femelles appariés, et par comparaison des deux méthodes.

**Résultats:**

Quarante rats mâles adultes sexuellement matures ont été sélectionnés et répartis aléatoirement dansquatre groupes groupe témoin (*n* = 10), groupe d’opération simulée (*n* = 10), groupe d’amputation unilatérale du MIC (Uni-MIC) (*n* = 10), et groupe d’amputation bilatérale du MIC (Bi-MIC) (*n* = 10). Quatre-vingts rats femelles en période de reproduction ont été réparties de façon aléatoire dans chacun des groupes mentionnés ci-dessus, selon un rapport de 2:1. Nous avons évalué le temps avant la conception des rats femelles appariés et les effets de l’amputation Uni /Bi-MIC sur la fonction érectile. Les résultats ont montré que la pression de base et la PIC maximale dans le groupe témoin, le groupe d’opération simulée, le groupe Uni-MIC et le groupe Bi-MIC étaient respectivement de 17.44 ± 2.50 mmHg et 93.51 ± 10.78 mmHg, 17.81 ± 2.81 mmHg et 95.07 ± 10.40 mmHg, 16.73 ± 2.11 mmHg et 83.49 ± 12.38 mmHg, et 14.78 ± 2.78 mmHg et 33.57 ± 6.72 mmHg, immédiatement après l’opération. La PIC maximale dans le groupe Bi-MIC était inférieure à celles des trois autres groupes (tous *P* < 0.05). Les taux de grossesse étaient de 90% dans le groupe Uni-MIC et de 0% dans le groupe Bi-MIC. Le taux de grossesse dans le groupe Bi-MIC était significativement plus faible que dans les autres groupes (tous *P* < 0.05). Le temps de conception a été d’environ 7 à 10 jours plus tard dans le groupe Uni-MIC que dans le groupe témoin et le groupe d’opération simulée (tous *P* < 0.05).

**Conclusions:**

Les rats mâles subissant une amputation Bi-MIC peuvent développer une DE permanente qui affecte leur fertilité. En revanche, ceux qui subissent une amputation Uni-MIC peuvent connaître des troubles érectiles transitoires.

## Background

The ischiocavernosus muscle (ICM) encompasses a pair of short pinnate muscles attached to the pelvic ring. This muscle originates at the ischial tuberosity and ends at the crus of the penis while covering the surface of the crus [1–4]. According to the traditional view, the contraction of the ICM acts as a lever on the upturned penis and plays an auxiliary role in penile erection [4]. However, our previous studies reported on the role of ICM contractions in preventing blood backflow into the corpus cavernosum and maintaining a stiff erection [5, 6]. Moreover, findings from the series of our basic studies on the ICM suggested that unilateral amputation of the ICM in a male rat could cause transient erectile dysfunction (ED), thus resulting in delayed conception in the paired female rat. In contrast, bilateral amputation of the ICM in a male rat could cause permanent ED, thereby resulting in an inability to conceive in the paired female rat [5]. Erectile function improved in some male rats following the repair of the severed ICM, which offered their female mates the ability to conceive. Furthermore, we confirmed the existence of objective evidence on an external force-mediated ICM injury in the pelvic fracture model in male rats, but there was relatively little evidence of vascular and nerve injuries related to factors causing ED [5].

In addition, in clinical practice, we performed computed tomography (CT)/magnetic resonance imaging (MRI)/electromyography (EMG) for patients with pelvic fracture with ED as a complication and found objective evidence of ICM injury [6]. Our previous studies provided novel ideas for the clinical diagnosis and treatment of ICM injury and ED following a pelvic fracture [5, 6].

The diagnosis of ED often depends on measuring the intracavernosal pressure (ICP) and sometimes on drug (such as apomorphine and sildenafil) or nerve stimulation [7–12]. Furthermore, ICP is the current gold standard for the diagnosis of ED [11–15]. Nonetheless, our indirect method of diagnosing ED with ICM injury by observing the infertility of paired female rats needs further validation [5].

Thus, our aim was to amputate the ICM, establish an ED model of ICM injury, record ICP, record infertility in matched female rats, and compare the two methods. This study will provide a certain degree of reference for the diagnosis of ICM injury and its treatment in clinical patients with pelvic fracture with ED as a complication.

## Materials and methods

### Experiment animals

We purchased 120 Sprague–Dawley rats (SPF degree; males and females) from the Experimental Animal Center of the Third Military Medical University. All animal operations were approved by the Animal Management Committee of Zunyi Medical University.

### Animal grouping

Male rats of reproductive age (*n* = 40, age=4–6 months, weight=200–300 g) were randomly assigned to four groups, namely, the unilateral ischiocavernosus muscle (Uni-ICM) amputation group, bilateral ischiocavernosus muscle (Bi-ICM) amputation group, sham operation group, and control group. Each experimental group consisted of 10 rats. Following the surgery, the male rats were separately mated with reproductively active females (*n* = 80, age=4–6 months, weight=200–300 g) to determine the severity of ED.

### Surgical equipment

The anesthetic comprised 5% amobarbital sodium, which was intraperitoneally injected at a dose of 1 ml/100 g. Furthermore, we used 0.5% povidone-iodine as a disinfectant. The general surgical tools included a vas-deferens-separating clamp, a pair of ophthalmic scissors, a pair of suture scissors, a needle holder, scalpels, two leather clamps, two forceps, four mosquito clamps, one small round needle, one triangular needle, #1 and #4 sutures, and microsurgical tools.

## Model establishment and observation

### Operation to expose the ICM

**Fig. 1:**
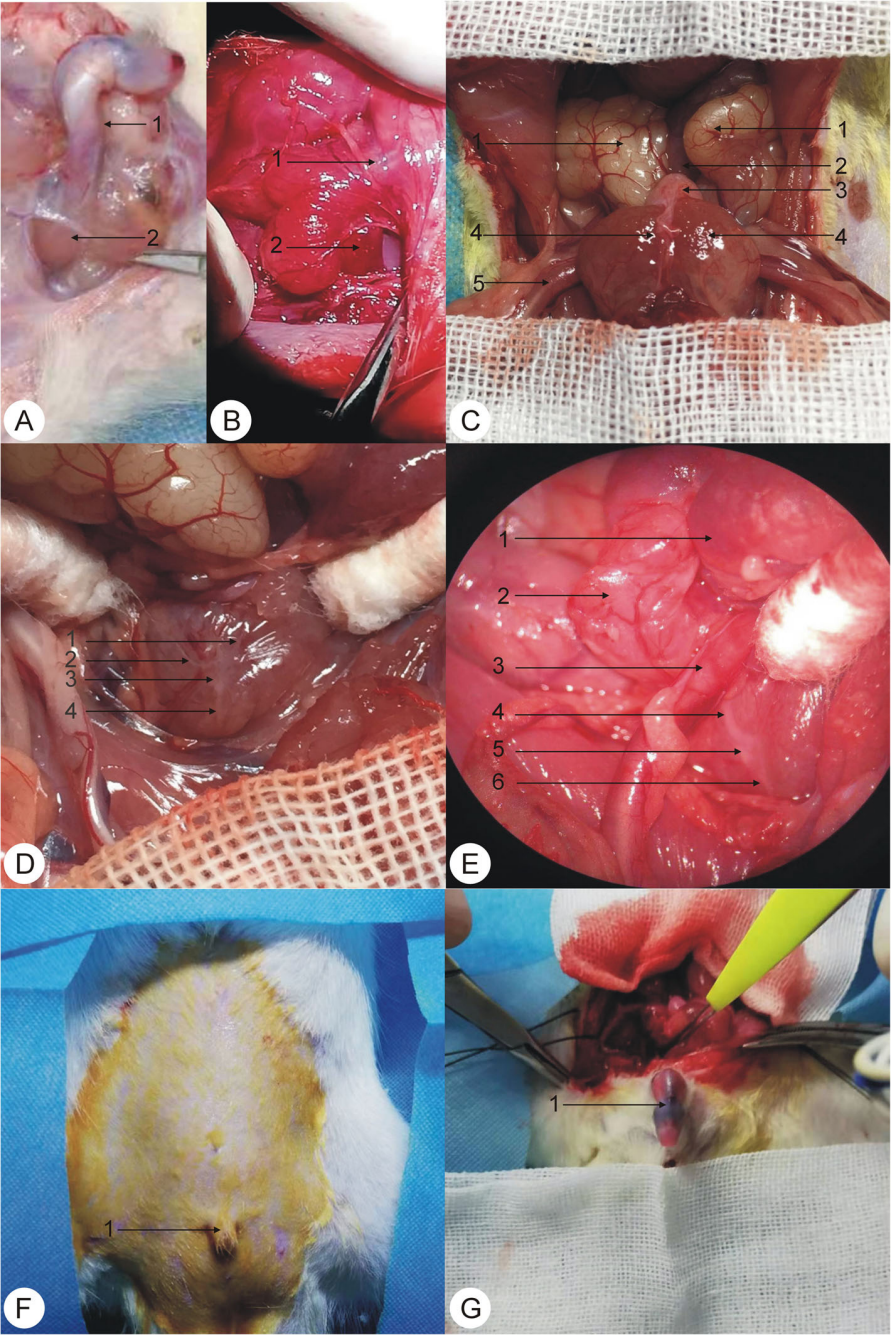
Exposure of the ischiocavernosus muscle and cavernous nerve during the surgery and the induction of penile erection by electrical stimulation of the cavernous nerve in male rats. A:1. the root of the penis and 2. ischiocavernosus muscle; B:1. penis and 2. ischiocavernosus muscle; C:1. seminal vesicle gland, 2. rectum, 3. bladder, 4. prostate gland, and 5. vas deferens; D:1. hypogastric nerve, 2. pelvic nerve, 3. pelvic ganglia, and 4. cavernous nerve; E:1. bladder, 2. seminal vesicle gland, 3. vas deferens, 4. hypogastric nerve, 5. pelvic ganglia, and 6. cavernous nerve; F:1. a nonerect penis of a male rat; G:1. an erect penis of a male rat after electrical stimulation

The rats in each experimental group were weighed, anesthetized, and placed onto an operating table. Using the principles of aseptic surgery, a straight incision was made in the perineum after both testicles were pushed into the abdominal cavity. The length of the incision was approximately 2 cm. The skin was cut open, and the subcutaneous fat and superficial perineal fascia were cut obtusely. We exposed both ischiocavernosus muscles at the perineum according to the method reported by Tong Liu et al. [5]. The ICM was cut 1 cm from the crus penis according to the corresponding surgical requirements of the respective group (Fig. 1A and B). We stipulated that the right ICM should be amputated uniformly in the Uni-ICM amputation group. The left and right ICMs were amputated in the Bi-ICM amputation group. Although we isolated the ICM in the sham operation group, it was not amputated. Rats in the control group did not undergo any surgical procedure.

### Operation to expose the cavernous nerve of the penis

The abdominal cavity was squeezed at the end of the ICM operation to reposition the testicles into the scrotum. We made an incision with a length of approximately 4 cm in the middle of the lower abdomen. The skin, subcutaneous tissue, and rectus abdominis were cut into the abdominal cavity, after which the incision was pulled to both sides. We pushed up the intestinal tube to completely expose the pelvic organs. This in turn facilitated viewing the seminal vesicle, rectum, bladder, prostate, and root of the penis from top to bottom. We used an assisted microsurgical technique (Leica M220 F12, Leica Microsystems, Germany) to separate, cut off, and ligate the lateral ligaments of the bladder under a microscope. This was done to expose the lateral back of the prostate, open the pelvic fascia, look for the pelvic nerve and pelvic ganglion (a stellate milky white structure), and find the largest branch of the pelvic nerve. The latter is the cavernous nerve of the penis. The cavernous nerves of the penis were found to run diagonally down both sides of the prostate, into the pelvic floor and along both sides of the urethra (Fig. 1C, D, and E).

### ICP measurement

Following the exposure of the nerve, we connected the BL-420F biological function experiment computer recording system (Chengdu Taimeng Technology Co., Ltd. China) to the computer. The pressure transducer is connected to channel 1, the two ends of the pressure transducer are connected with a three-way tube, and the transducer and the puncture needle are prefilled with saline containing heparin for calibration and zero adjustment. After zero adjustment, the needle was inserted into the dorsal side of the cavernosum of the penis, and the angle of insertion was kept at 10° and the depth of puncture was kept at 0.5 cm. Successful puncture was determined by injecting a small amount of saline containing heparin into the cavernosum of the penis to produce penile enlargement on the puncture side. After a successful puncture, the biological function experiment system was turned on, the pressure signal acquisition mode was set, and the computer displayed the baseline ICP of the cavernous body of the penis. Then, the bipolar hook was connected to the stimulation output end of the BL-420F instrument, and the stimulation parameters were selected for a good induced erection waveform (stimulation intensity: 5 V; wave width: 2 ms; frequency: 24 Hz; and stimulation time: 1 min).

We recorded the ICP during electrical stimulation in real time. The max ICP was stabilized (at least 4 min) at the end of the single stimulation. We selected the cavernosum pressure before electrical stimulation as the baseline ICP value. In contrast, the highest value of the cavernosum pressure during the entire stimulation was selected as the max ICP value. Electrical stimulation of the cavernosum nerve facilitated the continuous recording of the ICP, including the baseline and max ICP, three times.

Following the completion of the recording, 125,000 long-acting penicillin units were injected intraperitoneally, and perineal incisions were made to prevent infection. Dezocine 1.25 mg·kg^−1^ was injected intraperitoneally to alleviate pain postsurgery. The layers of the abdominal cavity and perineal incision were closed using no. 1 silk thread. We disinfected the surgical site with iodophor. We remeasured the baseline and max ICP of each group 2 months after the first measurement.

Male and female rats were matched, and pregnancy was recorded.

Each male rat was fed separately for 1 week and paired with female rats with a male-to-female ratio of 1:2 following the first measurement. We placed the rats in a quiet dark room and provided them compound food and drinking water. Pregnancy was recorded in paired female rats, and the birth of female rats was used as a marker to judge the mating success of male rats. We continuously observed the pregnancy for 2 months and maintained daily records.

The female and male rats were killed at the end of our experiments by cervical dislocation.

## Standard of erectile function in male rats

### Determination of erectile function in male rats by the penile sponge body pressure test

Electrical stimulation of the cavernous nerve of the penis resulted in the swelling of the corpus cavernosum, with the penis turned up at an angle >90°. We measured the max ICP in the corpus cavernosum, which represented normal penile erection function. The upward angle of the penis was <90° with no obvious swelling of the corpus cavernosum. We measured the max ICP, which represented penile ED (Fig. 1F and G).

### Determination of erectile function in male rats according to the pregnancy status of the paired females

The male rats were bred with female rats, and their erectile function was inferred indirectly based on the fertilization of the female rats. We examined the presence of external vaginal emboli in female rats and subsequent births by female rats as markers of fertilization in female rats. In addition, the failure of the paired female rats to conceive indicated ED in the male rats. Delayed conception of the female rats indicated temporary ED in their male counterparts. Furthermore, successful pregnancy of the female rats at a normal time indicated the absence of ED in the male rats. The normal erectile function and fertility of the male and paired female rats, respectively, were taken as references.

### Statistical analyses

Statistical analyses were performed using the Statistical Package for the Social Sciences (SPSS Inc., Chicago, USA) version 18.0 for Windows (SPSS Inc., Chicago, IL, USA). The ICP values, including the baseline and max ICP, and the time to conception were recorded as the mean±standard deviation, and the pregnancy rate of the male rats was recorded as a percentage. One-way analysis of variance and t tests were used to compare the differences in quantitative data among groups. Moreover, covariance analysis was used to analyze the statistical significance of the differences in max ICP following the adjustment of baseline ICP. The chi-square test was used to compare the pregnancy rate of the paired female rats in each group. A *P* value <0.05 was considered to indicate statistical significance.

## Results

### Death of male rats

One rat died in the control group, 6 days postoperatively; one rat died in the sham operation group, 9 days postoperatively; no deaths occurred in the Uni-ICM amputation group; and two died in the Bi-ICM amputation group, 1 and 2 days postoperatively.

### Baseline and max ICP measured immediately after the operation

The baseline and max ICP in the control group, sham operation group, Uni-ICM amputation group, and Bi-ICM amputation group were 17.44±2.50 mmHg and 93.51±10.78 mmHg, 17.81±2.81 mmHg and 95.07±10.40 mmHg, 16.73±2.11 mmHg and 83.49±12.38 mmHg, and 14.78±2.78 mmHg and 33.57±6.72 mmHg, respectively, immediately postsurgery. There were significant differences between the baseline and max ICP in the male rats in each group (*P*<0.001) (Table 1). There was no significant difference in the baseline ICP among the four groups (*P*>0.05). Following the adjustment of the baseline ICP of the male rats in each group by covariance analysis, the differences in the max ICP of the male rats in each group were statistically significant (*F* = 79.691, *P*<0.001). Pairwise comparisons revealed statistically significant differences between the Bi-ICM amputation and control groups, Bi-ICM amputation and sham groups, and Bi-ICM amputation and Uni-ICM amputation groups (all *P*<0.05). Nonetheless, there was no substantial difference in the max ICP among the Uni-ICM amputation group, sham operation group, and control group (all *P*>0.05).

**Table 1:** Comparison of the intracavernosal pressure changes induced by electrical stimulation of the cavernous nerves in rats measured immediately after surgery

Group	Control group (*n* = 10)	Sham operation group (*n* = 10)	Uni-ICM amputation group (*n* = 10)	Bi-ICM amputation group (*n* = 10)	*F*	^1^*P* value
Baseline ICP (M±SD, mmHg)	17.44±2.50	17.81±2.81	16.73±2.11	14.78±2.78	2.833	0.052
Max ICP (M±SD, mmHg)	93.51±10.78	95.07±10.40	83.49±12.38	33.57±6.72*	79.691	**<0.001**
*t*	-21.74	-22.67	-16.81	-8.17		
^2^*P* value	**<0.001**	**<0.001**	**<0.001**	**<0.001**		

*Uni-ICM* unilateral ischiocavernosus muscle, *Bi-ICM* bilateral ischiocavernosus muscle, *ICP* intracavernosal pressure, *M±SD* mean±standard deviation

^1^*P value*s were calculated using one-way analysis of variance; the Bonferroni t test was used for multiple comparisons

^2^*P value*s were calculated using covariance analysis of max ICP, adjusted for the baseline ICP

* indicates that the max ICP of the Bi-ICM amputation group was significantly lower than those of the control group, sham operation group, and Uni-ICM amputation group (all *P*<0.05)

Significant at *P*<0.05

### Baseline and max ICP measured 2 months after the operation

The baseline and max ICP in the control group, sham operation group, Uni-ICM amputation group, and Bi-ICM amputation group were 16.82±3.07 mmHg and 90.91±8.04 mmHg, 18.44±2.80 mmHg and 91.40±10.33 mmHg, 15.72±4.27 mmHg and 89.06±9.87 mmHg, and 17.98±2.93 mmHg and 59.12±5.88 mmHg, respectively, 2 months postoperatively. There were significant differences in the baseline and max ICP in each group (*P*<0.001) (Table 2). There was no significant difference in the baseline ICP among the male rats 2 months postoperatively (*P*>0.05). Following the adjustment of the baseline ICP of the male rats in each group by covariance analysis, the differences in the max ICP of the male rats were statistically significant (*F* = 26.22, *P*<0.001). Pairwise comparisons revealed statistically significant differences between the Bi-ICM amputation and normal groups, Bi-ICM amputation and sham operation groups, and Bi-ICM amputation and Uni-ICM amputation groups (all *P*<0.05). Furthermore, there was no significant difference in the Max ICP among the control group, sham operation group, and Uni-ICM amputation group (*P*>0.05).

**Table 2:** Comparison of the intracavernosal pressure changes induced by electrical stimulation of the cavernous nerves in rats 2 months postoperatively

Group	Control group (n = 9)	Sham operation group (*n* = 9)	Uni-ICM amputation group (*n* = 10)	Bi-ICM amputation group (*n* = 8)	*F*	^1^*P* value
Baseline ICP (M±SD, mmHg)	16.82±3.07	18.44±2.80	15.72±4.27	17.98±2.93	1.233	0.314
Max ICP (M±SD, mmHg)	90.91±8.04	91.40±10.33	89.06±9.87	59.12±5.88*	26.22	**<0.001**
*t*	-25.83	-20.46	-21.57	-17.71		
^2^*P* value	**<0.001**	**<0.001**	**<0.001**	**<0.001**		

*Uni-ICM* unilateral ischiocavernosus muscle, *Bi-ICM* bilateral ischiocavernosus muscle, *ICP* intracavernosal pressure, *M±SD* mean±standard deviation

^1^*P value*s were calculated using one-way analysis of variance; the Bonferroni t test was used for multiple comparisons

^2^*P value*s were calculated using covariance analysis of the max ICP, adjusted for the baseline ICP

* indicates that the Max ICP of the Bi-ICM amputation group was lower than those of the control group, sham operation group, and Uni-ICM amputation group, but the difference was statistically insignificant (all *P*<0.05)

Significant at *P*<0.05

### Pregnancy rate and the time to conception in successfully mated female rats

There were nine control group matched paired females with a pregnancy rate of 100% (Table 3). Likewise, there were nine paired females in the sham operation group with a pregnancy rate of 100%. In contrast, the pregnancy rate was 90% for the 10 paired females in the Uni-ICM amputation group. Nonetheless, the pregnancy rate was zero for the eight paired females in the Bi-ICM amputation group. The time to conception was 21±2 days, 23±3 days, and 29±6 days in the control group, sham operation group, and Uni-ICM amputation group, respectively, and with no conception in the Bi-ICM amputation group. The differences in the pregnancy rate (*x*^2^=89.53, *P*<0.001) and time to conception (*F* = 111.09, *P*<0.001) in each group were statistically significant. Pairwise comparison revealed no significant difference in the pregnancy rate among the Uni-ICM amputation group, control group, and sham operation group (*P*>0.05). The pregnancy rate in the Bi-ICM amputation group was significantly lower than that in the control group, sham operation group, and Uni-ICM amputation group (all *P*<0.05). In addition, the time to conception was delayed in the Uni-ICM amputation group compared with the control group and sham operation group (all *P*<0.05).

**Table 3:** Comparison of the cohabiting conception rate and time to conception for paired rats in the control group, sham group, UNI-ICM group and BI-ICM group

Group	Number of successful matches	Number of pregnancies obtained	Number of failed pregnancies	^**1**^Time to conception (M±SD, days)	^**2**^Pregnancy (%)	*F*/χ^2^	*P* value
Control group	9	9	0	21±2	100*	111.09/89.53	**<0.001**
Sham operation group	9	9	0	23±3	100		
Uni-ICM amputation group	10	9	1	29±6^Φ^	90^#^		
Bi-ICM amputation group	8	0	8	*No*	0^Δ^		

*Uni-ICM* unilateral ischiocavernosus muscle, *Bi-ICM* bilateral ischiocavernosus muscle, *ICP* intracavernosal pressure, *M±SD* mean±standard deviation

^1^one-way analysis of variance; the Bonferroni t test was used for multiple comparisons

Φ indicates a statistically significant difference in the time to conception in the Uni-ICM amputation group compared to the control group and sham operation group (*P*<0.05)

* indicates there was no statistically insignificant difference in the pregnancy rate between the control group, sham operation group and the Uni-ICM amputation group (all *P*>0.05)

^2^Chi^2^ test, and the Fisher’s exact method calculates the probability directly

# indicates a statistical significance difference in the pregnancy rate between the Uni-ICM amputation and Bi-ICM amputation groups (*P*<0.05)

Significant at *P*<0.05

Δ indicates a statistically significant difference in the pregnancy rate in the Bi-ICM amputation group compared to those in the Uni-ICM amputation group, control group, and sham operation group (*P*<0.05)

## Discussion

We evaluated the erectile function of male rats and the pregnancy rate of their paired female mates by amputating the unilateral/bilateral ICM. Our results showed that the max ICP of the Bi-ICM amputation group was significantly lower than those of the Uni-ICM amputation group, control group, and sham operation group. Furthermore, the penis could not be effectively erect in the Bi-ICM amputation group. However, the max ICP of the Uni-ICM amputation group was not significantly reduced compared to that of the control group and sham operation group. At the same time, the penis could be effectively erected in the Uni-ICM amputation group. While the pregnancy rate of the paired female rats in the Bi-ICM amputation group was zero, it was 90% in the Uni-ICM amputation group and 100% in both the control and sham operation groups. Clearly, the pregnancy rate in the Bi-ICM amputation group was lower than that in the Uni-ICM amputation group, control group, and sham operation group. Furthermore, the difference in the pregnancy rate in the Uni-ICM amputation group was not obvious compared to that in the control and sham operation groups. Thus, we attributed the reduction in the pregnancy rate in the Bi-ICM amputation group to ED.

According to the literature, there are two views on the role of the ICM in the mechanism of penile erection. One view was that ICM plays only an auxiliary role in the mechanism of penile erection [1–4]. However, another view, including the study authors’, was that ICM plays an important role in the mechanism of penile erection [5, 6, 14–18]. The results of this study also support the latter view.

Holmes GM et al. reported that ICM EMG showed that erection and penis insertion into the vagina were accompanied by sustained and intense contraction of the ICM during copulation in male rats [19]. Hart BL reported that surgical removal of the ICM reduced the occurrence of penile flips and virtually eliminated long or extended flips, and intromission or ejaculation patterns were absent [20]. Sachs BD reported that when the ICMs of rats were excised, no dorsiflexions (‘flips’) of the glans penis occurred during ex copula reflex tests. In attempted copulation, males lacking an ICM rarely gained intromission, apparently because the dorsiflexion of the glans penis is necessary for the penetration of the vagina [21]. Purohit RC et al. reported that ICM anesthesia with lidocaine significantly reduced the corpus cavernosum penis pressure, and dogs with low pressures were unable to copulate because of insufficient erection for intromission, which indicated that the ICM was the source of energy for the high corpus cavernosum penis pressure [22]. Based on the above analysis of the penile erection mechanism, we found evidence that supported that when sexual stimulation or sexual behavior occurs, the main role of the ICM might lie in the contraction of the ICM, which blocks blood backflow into the cavernosa and maintains a stiff erection, which is good for penis insertion into vagina and helps ejaculation [19–22]. When bilateral ICM injury occurred, it may have led to ED, and intromission or ejaculation patterns were absent, resulting in unsuccessful copulation and inability to fertilize, resulting in the partner being unable to become pregnant [16, 17, 19, 20].

In this experiment, we also observed that the duration of the max ICP of the Uni-ICM amputation group was shorter than that of the control and sham operation groups immediately postsurgery. Moreover, the max ICP of the Uni-ICM amputation group was slightly lower than those of the control and sham operation groups. The time to conception for the paired female rats was approximately 7–10 days later in the Uni-ICM amputation group (29±6 days) than in the control group (21±2 days) and sham group (23±3 days). Therefore, we speculate that male rats undergoing unilateral ICM amputation might experience transient ED. In addition, we observed a pairing without pregnancy in the Uni-ICM amputation group. Upon dissecting the male rat, we found pelvic inflammation, tissue and organ adhesion, and bilateral inflammation in the ejaculatory tubes. The cause of sterility may have been related to the postoperative infection and ejaculatory duct inflammation caused by the surgery.

In this study, 4 rats died, including 1 rat in the control group. We analyzed the main cause of death as postoperative infection, with postoperative bleeding in one rat, although we performed the surgery strictly following aseptic principles and administered antibiotics to prevent infection postoperatively. The occurrence of infection may have been related to the low cleanliness level of the feeding environment, and the possibility of a nonstandard operation procedure cannot be ruled out. Thus, our attention is required in terms of surgical practices and improvements in the cleanliness level of the feeding environment.

We used t tests (not shown in the results section) to compare the max ICP recorded immediately after surgery with the max ICP recorded 2 months after surgery in each group. There was a certain degree of increase in the unilateral ICM amputation group (83.49±12.38 vs. 89.06±9.87 mmHg) and the bilateral ICM amputation group (33.57±6.72 vs. 59.12±5.88 mmHg), and the differences were statistically significant (all *P*<0.001). Interestingly, ED did not appear in the unilateral ICM amputation group. However, ED was always present in the bilateral ICM amputation group. Therefore, we concluded that both ICMs play an interrelated role in the penile erection mechanism. This interaction occurs because the ICM has interconnecting partial crossing fibers at the base of the penis, and this structure, in our previous studies, was named the “chiasm of crus penis” of the ICM and will be further introduced in subsequent studies.

In this study, we used two methods to evaluate ED caused by ICM injury, monitoring ICP and recording the pregnancy of paired female rats, and the results of the two methods were consistent. Breeding and recording the pregnancy rate is a simple, economical and noninvasive method that deserves to be promoted.

However, our study had some limitations. We adopted a mating method to indirectly evaluate the erectile function of male rats by observing the pregnancy of their paired females [5] instead of the internationally recognized ICP method [11–16]. This method is not suitable for male or female rats with internal reproductive organ problems. Furthermore, the impact of species differences on the application of our findings to humans requires clarification [6, 23–29].

## Conclusion

In our study, unilateral ICM amputation may not affect erectile function in male rats or cause only transient ED, and it does not affect fertility. However, bilateral ICM amputation may cause ED in male rats and affect their fertility. Both ICMs play an interrelated role in the penile erection mechanism.

## Data Availability

The datasets generated and/or analyzed during the current study are available from the corresponding author upon reasonable request.

## Abbreviations

ED: Erectile dysfunction
ICM: Ischiocavernosus muscle
ICP: Intracavernosal pressure
Bi-ICM: Bilateral ischiocavernosus muscle
Uni-ICM: Unilateral ischiocavernosus muscle
cm: Centimeter
g: Gram
kg: Kilogram
min: Minute
Hz: Hertz
ml: Milliliter
mm: Millimeter
mmHg: Millimeter of mercury
ms: Millisecond
V: Volt
